# Effect of periodontal therapy with systemic antimicrobials on parameters of metabolic syndrome: A randomized clinical trial

**DOI:** 10.1111/jcpe.12763

**Published:** 2017-07-12

**Authors:** Sergio Bizzarro, Ubele van der Velden, Wijnand J. Teeuw, Victor E. A. Gerdes, Bruno G. Loos

**Affiliations:** ^1^ Department of Periodontology Academic Centre for Dentistry Amsterdam (ACTA) University of Amsterdam and Vrije Universiteit Amsterdam Amsterdam The Netherlands; ^2^ Department of Internal Medicine MC Slotervaart Amsterdam The Netherlands

**Keywords:** antimicrobials, metabolic syndrome, periodontal therapy, periodontitis, systolic blood pressure, triglycerides

## Abstract

**Aim:**

To investigate the effect of basic periodontal therapy (BPT) with antimicrobials (AM) on the parameters of metabolic syndrome (MetS) (waist circumference, systolic/diastolic blood pressure [BP], HDL‐cholesterol, triglycerides, glucose).

**Methods:**

One hundred and ten periodontitis patients without known comorbidities and unaware of possible MetS were randomly assigned to BPT (*n* = 56) or BPT+AM (*n* = 54) and followed for 12 months post‐therapy. Number of patients with undiagnosed MetS was also determined.

**Results:**

In all patients, the periodontal condition improved; however, the BTP+AM group showed greater pocket depth reduction than the BPT group. Post‐therapy, systolic BP (*p *<* *.05) and triglycerides (*p *<* *.05) reduced significantly during the follow‐up period. No significant differences could be assessed between the BPT+AM and BPT group. Despite the absence of self‐reported comorbidities, 27.2% (*n* = 30) periodontitis patients fulfilled the criteria of MetS at baseline. After therapy, this proportion changed to 14.5% at 3 months (*p *=* *.007), to 17.3% at 6 months (*p *=* *.017) and to 21.8% at 12‐month follow‐up (*p *=* *.383).

**Conclusion:**

Although a reduction in systolic BP and triglycerides and a temporarily improvement in the whole metabolic status were observed, the use of antimicrobials in conjunction with BTP does not yield any additional improvement in the parameters of MetS.

## 1 INTRODUCTION

Metabolic syndrome (MetS) is a combination of metabolic disturbances defined by central obesity and any of two of the following additional factors: hypertension, raised fasting plasma glucose and dyslipidaemia, that is raised triglycerides or reduced high‐density lipoproteins (HDL) (Alberti, Zimmet, & Shaw, 2006). MetS is acquiring attention as a systemic condition as it can increase the risk of cardiovascular diseases (CVD) by twofold and diabetes mellitus type 2 (T2DM) by fivefold (Alberti et al., 2009; Gami et al., 2007), but it remains often undiagnosed.

Periodontitis is an inflammatory disease that may increase the risk of CVD (Tonetti & Van Dyke, 2013) and can negatively affect glycaemic control of diabetic patients (Lalla & Papapanou, 2011; Preshaw et al., 2012). It has been calculated that patients affected by moderate to severe periodontitis have periodontal ulcerated lesions (pockets) with a periodontal inflamed surface area (PISA) between 8 and 20 cm^2^ (Loos, 2005; Nesse et al., 2008). Through this ulcerated pocket wall, bacterial pathogens and lipopolysaccharides cause daily short‐lived bactaeraemias and endotoxaemias, which result in an increase in the systemic inflammatory burden (Bahrani‐Mougeot et al., 2008; Lockhart et al., 2008). Two reviews from a joint workshop by the European Federation of Periodontology and the American Academy of Periodontology confirmed that the pro‐inflammatory state in periodontitis can contribute to an increased risk of insulin resistance, and subsequently T2DM and CVD (Schenkein & Loos, 2013; Taylor, Preshaw, & Lalla, 2013).

Although there is substantial evidence about the associations between periodontitis on the one hand, and T2DM and CVD on the other hand, evidence supporting a relationship between periodontitis and MetS is limited. Cross‐sectional studies showed an increase in the prevalence of MetS in periodontitis patients in comparison with individuals with gingivitis or healthy controls (Nibali et al., 2013; Tu, D'Aiuto, Lin, Chen, & Chien, 2013).

To date, several clinical trials investigated the effect of periodontal therapy on a variety of markers for the risk of CVD, both in patients with or without known comorbidities (Teeuw et al., 2014). These included mainly markers of systemic inflammation, dyslipidaemia, glucose and hypertension. However studies focusing specifically on the parameters of MetS, after periodontal treatment, are scarce and not congruent in their conclusions (Acharya, Bhavsar, Jadav, & Parikh, 2010; Lopez et al., 2012; Torumtay et al., 2016).

It has been suggested that the adjunctive use of systemic antimicrobials to basic periodontal therapy (BPT) may have a larger impact on markers of systemic inflammation (Demmer et al., 2013) than the BPT alone. In a previous publication, we showed that the combined use of systemic antimicrobials (amoxicillin and metronidazole) (AM) and BPT had a significant additional effect on the reduction in periodontal inflammation when compared with BPT without AM (Bizzarro, Van der Velden, & Loos, 2016).

This study is an extension of the latter investigation and reports on the parameters of MetS that were also assessed. Thus, the aim of the present one‐year randomized controlled trial was to investigate the effect of BPT with adjunctive AM on the five defining parameters (waist circumference, triglycerides, blood pressure, HDL‐cholesterol and glucose) of MetS in a population with periodontitis without known comorbidities in comparison with BPT without AM. In addition, the proportion of patients fitting the diagnosis of MetS was monitored.

## 2 MATERIAL AND METHODS

### 2.1 Study population

Consecutive subjects, who were referred to the Department of Periodontology of the Academic Center for Dentistry Amsterdam (ACTA) for treatment of periodontitis in the period 2008–2013, were screened for eligibility. Specifically, patients were eligible if they were not having any known comorbidity apart from periodontitis. Patients were recruited if they fulfilled the following inclusion criteria: presence of chronic periodontitis, self‐reported good general health, not being aware of any form of diabetes, diagnosis of MetS, CVD, (auto)immune disease or any other systemic or metabolic disease, and not receiving any medication for hypertension, dyslipidaemia or hyperglycaemia. A periodontal case was defined if he or she had proximal clinical attachment loss of at least ≥3 mm in ≥2 non‐adjacent teeth (Tonetti & Claffey, 2005). For this study, a patient was included if he or she presented ≥30% alveolar bone loss at ≥2 teeth per quadrant and presence of ≥2 teeth per quadrant with periodontal pockets ≥5 mm with at least ≥3 mm of clinical attachment loss and at least 50% of all sites in the mouth with bleeding on probing (BOP). Exclusion criteria were as follows: regular use of medications, use of antimicrobials in the past 6 months, periodontal treatment in the last 2 years, pregnancy/lactation, presence of implants or orthodontic appliances and presence of <20 natural teeth. Subjects who agreed to participate in this research signed a written informed consent. For this randomized controlled clinical trial, participants were randomized in four treatment modalities (Bizzarro et al., 2016). Patients received either BPT alone or, in addition to this, AM (amoxicillin 375 mg and metronidazole 250 mg, both 3 times daily for 7 days) (Winkel, Van Winkelhoff, Timmerman, Van der Velden, & Van der Weijden, 2001) and/or a single episode of subgingival disinfection with 0.5% sodium hypochlorite (NaOCl) (DIS) or saline (S). Examiner and therapists were blinded for patient allocation, and patients were blinded for the use of the NaOCl during therapy but not for the use of AM. There was therefore no placebo given to the patients allocated in treatment without AM. For the aim of this study, those patients allocated into the BPT+S and BPT+DIS groups were merged in one group of subjects who received BPT without AM (BPT), as the disinfection with NaOCl did not show any additional effect on the periodontal parameters (Bizzarro et al., 2016). Those allocated originally in the BPT+DIS+AM and BPT+S+AM groups were merged in one group who received the BPT with AM (BPT+AM). The study protocol was approved by the Medical Ethical Committee of the Academic Medical Centre of Amsterdam, the Netherlands (MEC 07/264). The research has been carried out in accordance with the Declaration of Helsinki (2013) of the World Medical Association. The protocol of this study has been retrospectively registered in Current Controlled Trials (ISRCTN36043780). The current manuscript followed the CONSORT guidelines.

### 2.2 General, medical and periodontal examinations

A recent and past medical history, demographic patient characteristics (ethnicity, age, education and gender), alcohol consumption and smoking habits were recorded by means of a questionnaire. A patient was defined as a smoker if he or she was currently smoking or quit ≤6 months before intake, and as a non‐smoker if he or she had never smoked or quit smoking >6 months before the baseline examination. Measurements for general health and MetS were taken: height and weight to calculate the body mass index (BMI) and the waist circumference (WC) at the umbilicus level, after exhaling. Blood pressure was measured between 09:00 and 11:00 AM, with a calibrated automated device (Omron M10‐IT, Omron Healthcare, Japan) at least 10 min after the patient quietly sat in the dental chair. The systolic (SBP) and diastolic (DBP) blood pressures were taken at both arms, and the mean of these measurements was calculated. Thereafter, fasting blood was collected, and periodontal parameters were recorded.

Periodontal assessments were performed at six sites per tooth and included dental plaque (presence/absence), BOP (presence/absence), probing pocket depth (PPD), clinical attachment level (CAL) and recessions (REC) after which the PISA was calculated (Nesse et al., 2008). All baseline measurements of general health and MetS as well as periodontal measurements were repeated at 3, 6 and 12 months after treatment by the same calibrated clinician (Bizzarro et al., 2016). Examiner reproducibility took place before the start of the study. In four subjects, full‐mouth‐duplicated measurements were recorded with a time interval of 30 min, and an intra‐class correlation was obtained for probing pocket depth (PPD) of 0.94 and for CAL of 0.95 (Bizzarro et al., 2016).

### 2.3 Periodontal therapy

The treatment protocol has been described in details in our previous publication (Bizzarro et al., 2016). In short, all patients received periodontal treatment, carried out by three experienced and specifically trained dental hygienists of the Department of Periodontology at ACTA in three appointments within 1 week. After completion of the active therapy, all patients were subsequently enrolled in a 3‐monthly maintenance programme at the Department of Periodontology until the end of the follow‐up (1 year) (Bizzarro et al., 2016).

### 2.4 Laboratory measures

After venipuncture, 0.5 ml of aliquots of EDTA plasma was prepared (after whole blood centrifugation, 2000 g for 10 min) and stored at −80ºC until further analysis. In one batch, HDL‐cholesterol, glucose and triglycerides were analysed with the enzymatic colorimetric methods on a modular analyzer Roche Cobas 8000 c502 (Roche Diagnostics Corp, Germany).

### 2.5 Statistical analysis

The power of the study, and consequently the number of study participants, was determined in our previous study based on clinical attachment level changes (Bizzarro et al., 2016) and not specifically based on expected outcomes for parameters of MetS. Statistical analysis was performed with a software package (SPSS 21.0, IBM Statistics, USA). Analysis was performed on an intention‐to‐treat basis (ITT). Data were explored with Little's Missing Completely At Random test (Groenwold, Donders, Roes, Harrell, & Moons, 2012). Missing data were imputed using the expectation–maximization method (Elashoff, Li, & Li, 2008). Outcomes were the defining parameters of MetS (WC, SBP/DBP, triglycerides, HDL, glucose). To test changes in the MetS outcomes after BPT, ANOVA for repeated measures was used with the AM usage as fixed factor and gender, age and smoking as covariates. These statistical tests yielded adjusted *p‐*values (*p*_adj_), and post hoc testing was performed with Bonferroni correction.

Criteria for diagnosis of MetS were based on the presence of central obesity (WC ≥102 cm in men or ≥88 cm in women) together with ≥2 of the following risk determinants: triglycerides ≥1.7 mmol/L, HDL <1.03 mmol/L in males or <1.29 mmol/L in females, blood pressure ≥130/85 mm Hg, fasting glucose ≥5.6 mmol/L (Grundy, 2008). The distribution of the patients with MetS (MetS+) or without (MetS‐) at baseline and at 3‐, 6‐ and 12‐month follow‐up was calculated for the two study groups. Intra‐group and inter‐group differences in prevalence were tested with McNemar test and with chi‐square test respectively, in both ITT and per‐protocol analyses.

*p *<* *.05 was considered statistically significant for the parametric statistics after Bonferroni correction where indicated. For the intra‐group analyses for the nonparametric data, *p‐*values ≤.017 were considered statistically significant (to correct for three pairwise comparisons).

## 3 RESULTS

**Figure 1:**
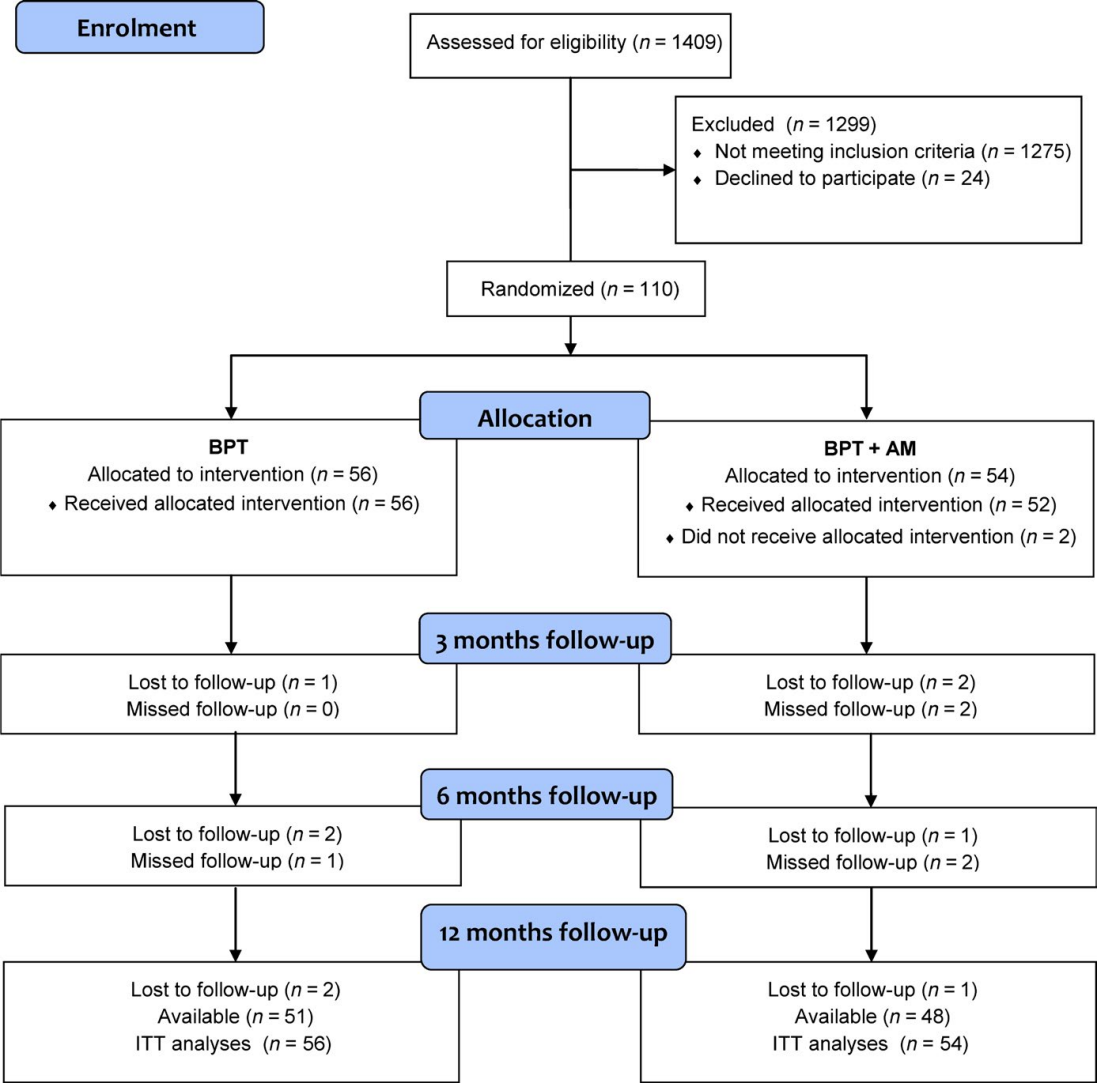
Flow chart of the study. Progress in patients enrolment, number of subjects who missed a follow‐up visit or dropped out are presented. A total of 1,275 subjects did not meet the inclusion criteria. Twenty‐four subjects did not wish to participate in the study. After treatment allocation, a total of 11 subjects did not attend the 12‐month follow‐up. One patient did not receive intervention because he did not show up at the appointments. One patient withdrew before treatment initiation. Nine patients dropped out because they did not want to attend the follow‐up visits. BPT, basic periodontal therapy; AM, antimicrobials; ITT, intention to treat

A total of 1,409 consecutive patients with periodontitis were screened, and 134 individuals met the inclusion criteria. The main reasons for not being eligible were having comorbidity, usage of any medication and not meeting periodontal case definition. Finally, 110 patients volunteered to participate in this study. At the end of the follow‐up, a total of 99 patients completed the study, and 11 patients (six in the BPT+AM group and five in the BPT group) were lost to follow‐up (Figure 1). Three patients (two in the BPT+AM group and one in the BPT group) started taking antihypertensive medication during the follow‐up. Exclusion of these patients did not change significantly the statistical results; therefore, these subjects have been retained in the final analyses.

The average age of the patients was 47.8 years, and the population was composed mainly of high‐educated, Dutch Caucasian patients. The average BMI was 25.2 kg/m^2^. There was a slight over‐representation of males and smokers, but no statistical differences were found between BPT and BPT+AM groups for any of the background variables (Table 1).

**Table 1:** Baseline characteristics of the study groups. Values are mean ± *SD* or number of subjects (%)

	BPT*N* = 56	BPT+AM^a^ *N* = 54
Age (years)	47.9 ± 9.4	47.6 ± 9.0
Gender		
Male	36 (64)	27 (50)
Female	20 (36)	27 (50)
Ethnicity		
Dutch Caucasian	41 (73)	40 (74)
Other	15 (27)	14 (26)
Education		
<High school	18 (32)	17 (31)
≥High school	38 (68)	37 (69)
Smoking		
Non‐smokers	27 (48)	21 (39)
Current smokers	29 (52)	33 (61)
Alcohol		
≥2 units/day	18 (32)	14 (26)
<2 units/day	38 (68)	40 (74)
BMI (kg/m^2^)	25.1 ± 3.0	25.3 ± 4.1

BMI, body mass index. BPT, basic periodontal therapy. AM, antimicrobials (amoxicillin 375 mg + metronidazole 250 mg, 3 x day x 7 days).

a No significant differences were observed for the baseline characteristics between BPT+AM and BPT (*p* > .05).

For the total study population, it was observed that periodontal therapy led to an improved periodontal condition through the whole follow‐up time. These results have been published before (Bizzarro et al., 2016). Data on PPD, CAL, REC, PISA, BOP and plaque are presented in the supplementary table (Table S1). In short, the BPT+AB group showed a significant additional improvement of the periodontal conditions in comparison with the BPT group, in particular, a greater decrease in PPD (mean difference 0.2 mm; [95% C.I. = 0.06, 0.42] *p*_adj_ <.05) and PISA (mean difference 2.31 cm^2^ [95% C.I. = 1.99,4.43] *p*_adj_ <.05) at all follow‐up time points.

### 3.1 Parameters of MetS

**Figure 2:**
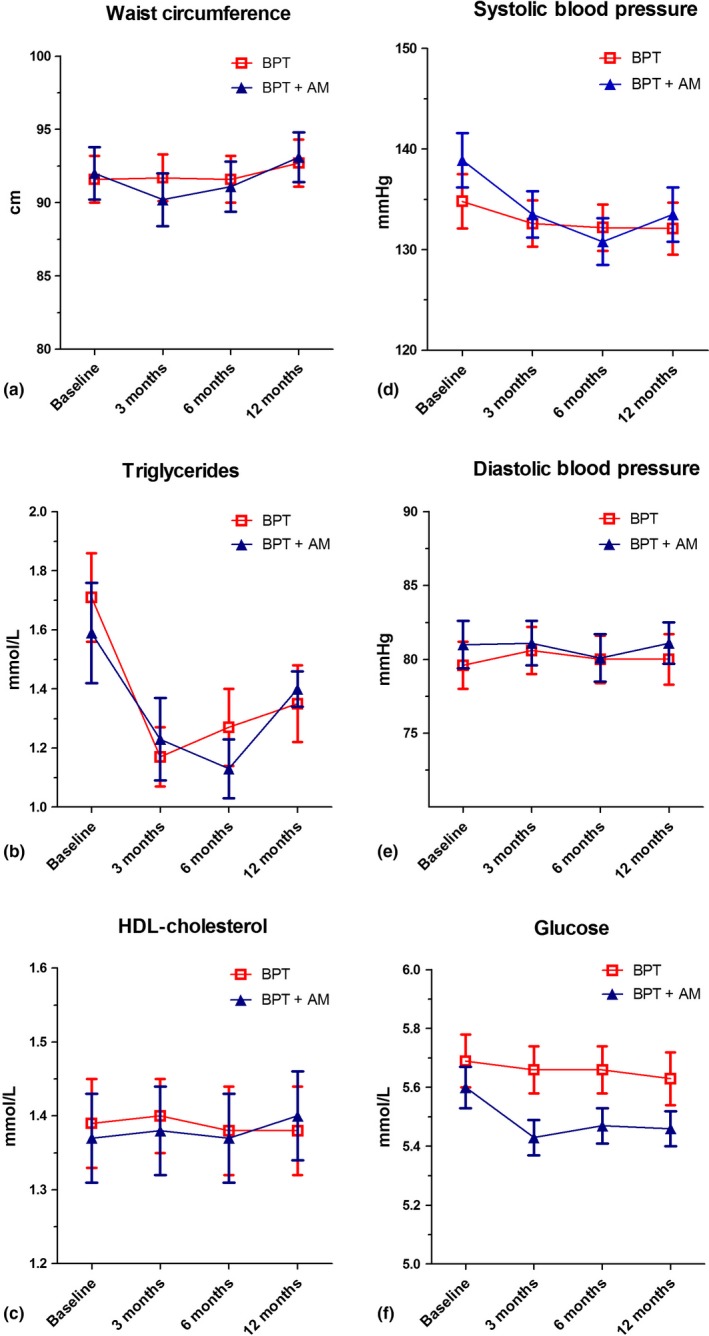
Changes of parameters of metabolic syndrome during 12 months follow‐up for BPT and BPT+AM groups. Changes for waist circumference (a), triglycerides (b), HDL‐cholesterol (c), systolic blood pressure (d), diastolic blood pressure (e) and glucose (f) are presented. Error bars represent standard errors of means. BPT, basic periodontal therapy; AM, antimicrobials

Figure 2 and Table S2 present the values of the parameters of MetS before and after therapy for the two study groups. At baseline, there were no significant differences for any of the five parameters of MetS between the BPT and the BPT+AM groups. After therapy, some parameters showed a statistical significant change overtime. In particular, at the 12‐month follow‐up visit, there were significant intra‐group reductions in SBP (from 134.8 to 132.1 mm Hg in the BPT group and from 138.9 to 133.5 mm Hg in the BPT+AB group, *p*_adj_ = 0.042) as well as triglycerides (from 1.71 to 1.35 mmol/L for the BPT group and from 1.59 to 1.28 mmol/L in the BPT+AB group, *p*_adj_ = 0.018) (Table S2). The inter‐group comparison showed no difference for these parameters (Figure 2). There were also significant intra‐group increases in WC at 12 months in comparison with 6 months (from 91.1 to 93.1 cm in the BPT group and from 91.6 to 92.7 cm in the BPT+AB group, *p*_adj_ = 0.027), but these increases were not significant different from the baseline values (92.0 and 91.6 cm in the BPT and BPT+AB group, respectively). The inter‐group analyses showed no significant difference for WC. No significant inter‐group and intra‐group differences were found for DBP, HDL‐cholesterol and fasting glucose (Figure 2 and Table S2).

### 3.2 Patients with diagnosis of MetS

At baseline, 30 patients (27.2%) (20 men and 10 females) unknowingly fulfilled the criteria of MetS (MetS+). The ITT analysis showed that for the total population, the number of MetS+ patients decreased to 16 (14.5%, *p *=* *.007) at the 3‐month follow‐up, but reverted to 19 patients (17.3%, *p *=* *.017) at 6‐month follow‐up and to 25 patients (21.8%, *p *=* *.383) at 12‐month follow‐up (Table 2). More specifically, at 12 months, from the 30 MetS+ periodontitis patients at baseline, 13 had no longer MetS (43.3% reduction), while nine patients (33%) changed their metabolic status only temporarily. However, from the 80 patients without MetS at baseline, eight (10%) showed to have MetS at follow‐up.

**Table 2:** Distribution of individuals with diagnosis of metabolic syndrome (MetS). Values are number of subjects (%)

	BPT		BPT+AM		Total	
	MetS+	MetS−	MetS+	MetS−	MetS+	MetS−
Baseline	14 (25.0)	42 (75.0)	16 (29.6)	38 (70.4)	30 (27.2)	80 (72.8)
3 months	10 (17.9)	46 (82.1)	6 (11.1)	48 (88.9)^c^	16 (14.5)	94 (85.5)^a^
6 months	11 (19.6)	45 (80.4)	7 (13.0)^c^	47 (87.0)^d^	19 (17.3)	91 (82.3)^b^
12 months	17 (30.4)	39 (69.6)	8 (14.8)	46 (85.2)^e^^,^ #	25 (21.8)	85 (78.2)

MetS+, patients with diagnosis of metabolic syndrome; MetS‐, patients without diagnosis of metabolic syndrome and see Table 1.

Based on intention‐to‐treat analysis. *p*‐values in comparison with baseline; ^a^ *p* = .007, ^b^ *p* = .017, ^c^ *p* = .013, ^d^ *p *= .022, ^e^ *p* = .039, (McNemar's test, *p* value ≤.017 was considered statistical significant, to correct for three pairwise comparisons). ^#^ *p* = .052 (chi‐square test) between BPT and BPT+AB groups. Diagnosis of metabolic syndrome: presence of central obesity (waist circumference ≥102 cm in men or ≥88 cm in women) and ≥2 of the following parameters: triglycerides ≥1.7 mmol/L, HDL‐cholesterol <1.03 mmol/L in men or <1.29 mmol/L in women, blood pressure ≥130/85 mm Hg, fasting glucose ≥5.6 mmol/L.

MetS+ patients who converted to MetS‐ were more prevalent in the BPT+AM group than in the BPT group (Table 2): from *N* = 16 at baseline to *N* = 6 at 3 months (*p *=* *.013), to *N* = 7 at 6 months (*p *=* *.022) and to *N* = 8 at 12 months after treatment (*p *=* *.039). Results of the inter‐group analyses showed a trend of a greater reduction in patients MetS+ in the BPT+ AM group in comparison with the BPT group (chi‐square test, *p *=* *.052). Data from per‐protocol analysis showed similar results, this is presented in Supplemental Tables S3 and S4, with frequencies of patients per time point of analysis.

## 4 DISCUSSION

The current randomized controlled clinical trial aimed to investigate the effect of BPT with the adjunct of AM on the five parameters of MetS in 110 patients with periodontitis, but without any self‐reported comorbidities. The clinical results showed that the use of AM yielded some extra improvements for the periodontal parameters, which remained stable throughout the total follow‐up. In particular, there was a greater reduction in the periodontal inflamed area in the BPT+AM group, as measured by the PISA, and a greater reduction in PPD. However, this increased reduction in the periodontal inflammation in the BPT+AM group was not associated with an adjunctive improvement in their metabolic condition. In fact, for none of the parameters of the MetS, a significant difference was found between the BPT+AM and BPT groups after treatment. However, for both groups, we found significant reductions in SBP and triglycerides over time. Considering the diagnosis MetS, a trend of a greater reduction in MetS+ patients after periodontal therapy with AM was present. These results might suggest that the adjunctive therapy of systemic AM to BPT could be more effective in improving the metabolic status of periodontitis patients with MetS. However, these results need to be interpreted as explorative as this is the result of an underpowered subanalysis.

It is important to note that the patients of the current study population were included as systemically healthy on the basis of an extensive medical history and self‐reported information. Nevertheless, 30 subjects of 110 (27.2%) fulfilled the criteria of MetS and were unaware of this condition. Although this investigation was not designed as a cross‐sectional or a population survey, it is interesting to note that the prevalence of MetS in our periodontitis population is higher than the prevalence reported previously in a cross‐sectional study in a Dutch population (Bos et al., 2007) and also higher than the prevalence of MetS reported in various populations in Europe (5%–27%) (Alberti et al., 2006). Interestingly, a recent investigation into hidden diabetes in periodontitis patients suggested that periodontitis is a possible early indicator of disturbed metabolic control (Teeuw, Kosho, Poland, Gerdes, & Loos, 2017). The current findings also point to the same direction and suggest that periodontitis patients may present with undiagnosed metabolic disturbances. Thus, general dentists, periodontists and dental hygienists need to be alert that severe periodontitis may be a symptom of an underlying systemic condition such as disturbed metabolic control. In such a case, it would be important to contact the general physician who can proceed with further specific diagnosis.

Considering the total study population, it has to be noted that a significant reduction in levels of triglycerides and SBP was observed after therapy. In addition, the percentage of patients who met the diagnosis of MetS was also, although temporarily, reduced after therapy. This suggests that the reduction in the periodontal inflammation may have had a beneficial impact on the metabolic condition of these subjects. This is in line with previous investigations which showed after periodontal therapy a reduction in markers of inflammation (D'Aiuto, Orlandi, & Gunsolley, 2013; Paraskevas, Huizinga, & Loos, 2008; Teeuw et al., 2014), markers of blood pressure such as endothelial function, flow‐mediated dilatation and arterial stiffness (Orlandi et al., 2014; Tonetti et al., 2007), and also an improvement in the lipid profiles (D'Aiuto et al., 2006; Mourao, Cataldo, Moutinho, Fischer, & Canabarro, 2014; Schenkein & Loos, 2013). We have to specify that, although in the main analyses, two of five parameters significantly decreased after therapy, the (temporary) improvement in the metabolic condition of patients with MetS was related to a decrease in single of a combination of parameters. At the 3‐month follow‐up, the conversion of MetS+ to MetS‐ (*n* = 18, Table S4) was caused in 12 patients by the reduction only in level of triglycerides (seven subjects) or reduction only in glucose (five subjects), while in six patients caused by a combination of three MetS parameters: reduction in glucose, triglycerides and SBP. In the supplemental material, a post hoc analysis is presented for patients diagnosed at baseline MetS+ and MetS‐ (Fig. S1). The analysis showed an inter‐group significant difference after therapy for the reduction in triglycerides (*p*_*adj*_ = 0.024). We noted also a decrease in levels of glucose and SBP, but due to the low number of subjects, there was no inter‐group significant difference (*p *=* *.437 for glucose and *p *=* *.100 for SBP).

A limitation of the current investigation is that the design does not allow an estimation of the impact of the reduction in the periodontal inflammation on the metabolic condition of these patients in relation to other possible confounders. In the current trial, an attempt has been made to note the most relevant factors which can influence the metabolic status. We did monitor changes in smoking habit and medication intake. Only a negligible number of patients showed a change in relation to these items. But we could not monitor favourable changes in diet or increased physical activity of these subjects, which are important interventions in the treatment of MetS (Stone, 2008). Nonetheless, for the total study population, we observed that an improved periodontal condition was associated with a reduction in SBP and lower levels of triglycerides. Moreover, for some individuals with periodontitis and MetS, a reversal to the negative status of MetS was noted.

It needs to be noted that the sample size for the current study was calculated on the basis of change in attachment level, which was the primary outcome of the original study protocol (Bizzarro et al., 2016) and not on the basis of changes in the parameters of MetS. Therefore, *stricto senso*, the current investigation needs to be labelled as a pilot study.

To test the objective impact of the periodontal therapy on patients' metabolic condition, an untreated control group would have been necessary. However, a real control group for this study would imply inclusion of patients with moderate to severe periodontitis without treatment for 1 year. Besides the fact that this is unethical and it is not allowed by the Dutch METC, it has been noted from previous periodontal intervention trials with >3‐month follow‐up, that a control group, composed of “community treated” or “untreated” subjects, suffers from a high drop‐out rate, a lack of compliance with the study protocol by seeking periodontal treatment outside the study (Couper et al., 2008; Lopez et al., 2012).

It has to be underlined that successful periodontal therapy requires highly compliant patients, motivated to perform on a daily basis an excellent oral hygiene. The compliance of the current population is reflected by the reduction in dental plaque, which can be considered as a measure of daily self‐performed care. It could be speculated that the active periodontal therapy and maintenance care given in the period of 1 year may have had also a positive influence towards their general health behaviour, particularly in relation to an improved diet and physical activity.

## 5 CONCLUSIONS

Based on the results of the current study, we can conclude that, although a reduction in systolic BP and triglycerides and a temporarily improvement in the whole metabolic status were observed, the use of antimicrobials in conjunction with BTP does not yield any additional improvement in the parameters of MetS.

## CONFLICT OF INTEREST

The authors have stated explicitly that there is no conflict of interests in connection with this article.

6

**Clinical Relevance**

*Scientific rationale for the study:* Periodontitis is associated with metabolic syndrome (MetS). Few studies investigated changes in the metabolic status in periodontitis patients after basic periodontal therapy (BPT). There are no data about the possible adjunctive effect of systemic antimicrobials (AM) in conjunction with BPT on the markers of MetS.

*Principal findings:* The use of systemic AM showed no additional effect on the parameters of MetS in comparison with the BPT alone, despite the fact that probing pocket depth and periodontal surface inflamed area were significant better. Regardless of the use of AM, a reduction in systolic blood pressure and triglycerides was observed after periodontal therapy.

*Practical implications:* Periodontal therapy may be beneficial for the improvement in the metabolic status; however, the use of systemic AM does not have any adjunctive effect for this purpose.
