# Supplementary figures and images for: Effect of periodontal therapy with systemic antimicrobials on parameters of metabolic syndrome: A randomized clinical trial
Source: J Clin Periodontol. 2017 Jul 12;44(8):833–41. doi: 10.1111/jcpe.12763 (PMC5599971; doi:10.1111/jcpe.12763)

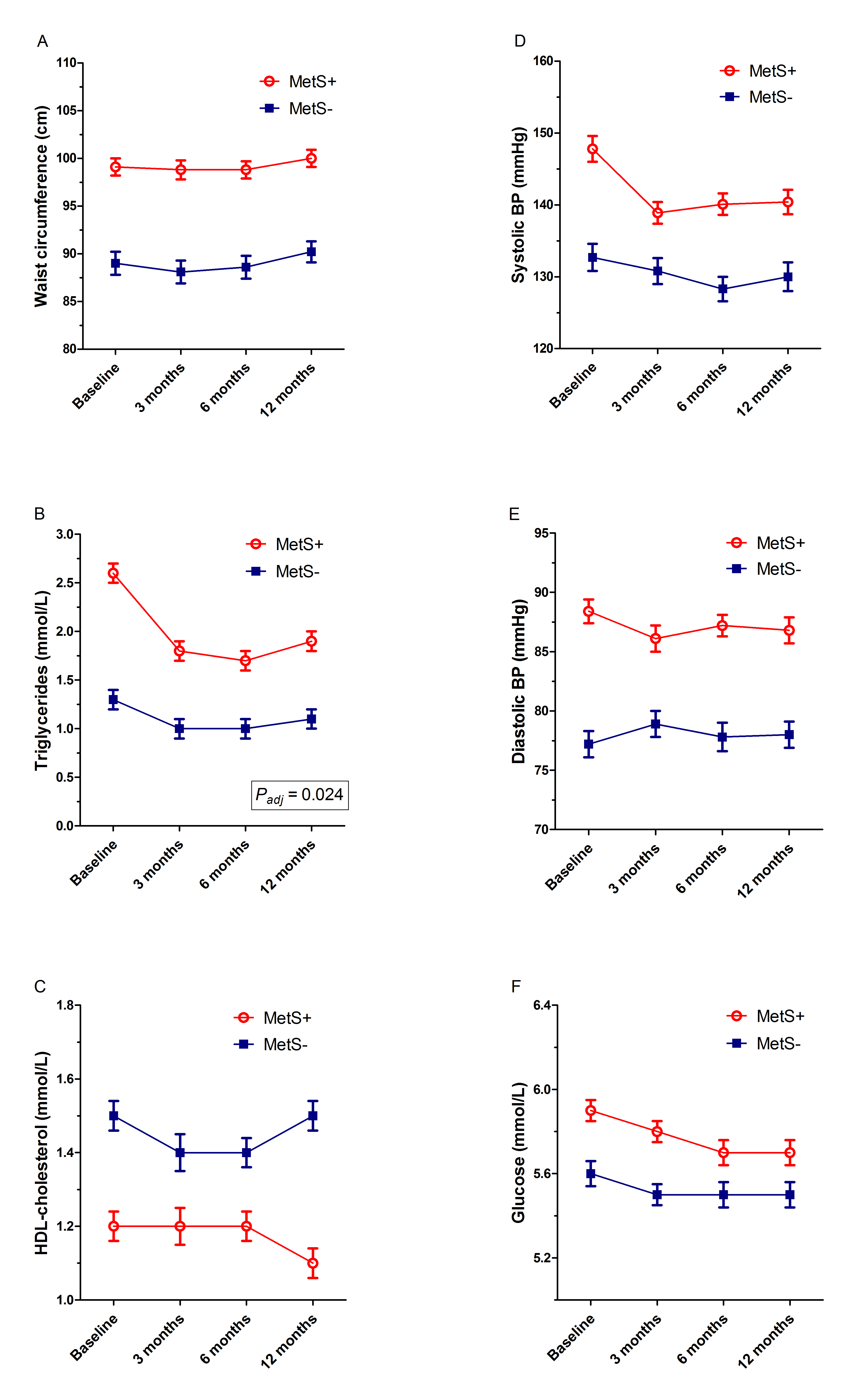

Supplement: Supplementary file 1 [file JCPE-44-833-s001.jpg]
