# Supplementary material for: Effect of periodontal therapy with systemic antimicrobials on parameters of metabolic syndrome: A randomized clinical trial
Source: J Clin Periodontol. 2017 Jul 12;44(8):833–41. doi: 10.1111/jcpe.12763 (PMC5599971; doi:10.1111/jcpe.12763)
Supplement: Supplementary file 2 [file JCPE-44-833-s002.docx]

**Effect of periodontal therapy with systemic antimicrobials on parameters of metabolic syndrome.**

**A randomized clinical trial**

Sergio Bizzarro^1^, Ubele van der Velden^1^, Wijnand J. Teeuw^1^, Victor E. A. Gerdes^2^, Bruno G. Loos^1^

^1^Department of Periodontology, Academic Centre for Dentistry Amsterdam (ACTA), University of Amsterdam and VU University Amsterdam, 1081LA, Amsterdam, the Netherlands.

^2^Department of Internal Medicine, MC Slotervaart, 1066EC, Amsterdam, the Netherlands.

**Supplementary material**

*Supplemental Table S1.* Effect of periodontal therapy on periodontal parameters. Values are means ± standard deviation.

|  |  |  |  | **BPT** |  | **BPT+AM** |  |
| --- | --- | --- | --- | --- | --- | --- | --- |
|  |  |  |  | N=56 |  | N=54 |  |
| PPD (mm) |  | Baseline  3 months  6 months  12 months |  | 3.8 ± 0.6 ^a^  2.9 ± 0.5  2.9 ± 0.5  2.9 ± 0.6 |  | 3.9 ± 0.7 ^a^  2.7 ± 0.4*  2.7 ± 0.4*  2.7 ± 0.5* |  |
| CAL (mm) |  | Baseline  3 months  6 months  12 months |  | 4.3 ± 0.8 ^a^  3.8 ± 1.0  3.8 ± 1.0  3.7 ± 1.0 |  | 4.2 ± 1.1 ^a^  3.5 ± 1.0  3.5 ± 1.0  3.5 ± 1.0 |  |
| REC (mm) |  | Baseline  3 months  6 months  12 months |  | 0.4 ± 0.7 ^a^  0.9 ± 0.9  0.9 ± 0.8  0.9 ± 0.8 |  | 0.3 ± 0.7 ^a^  0.7 ± 0.8  0.8 ± 0.8  0.8 ± 0.8 |  |
| BOP (%) |  | Baseline  3 months  6 months  12 months |  | 66.1 ± 14.8 ^a^  23.6 ± 13.2  23.7 ± 12.7  24.4 ± 14.7 |  | 68.7 ± 15.9 ^a^  20.3 ± 13.8  20.0 ± 14.9  22.0 ± 15.0 |  |
| PISA (cm^2^) |  | Baseline  3 months  6 months  12 months |  | 17.3 ± 4.9 ^a^  5.0 ± 3.4  5.0 ± 3.4  5.3 ± 4.0 |  | 18.3 ± 6.4 ^a^  3.6 ± 2.4*  3.7 ± 3.2*  4.0 ± 3.0* |  |
| Plaque (%) |  | Baseline  3 months  6 months  12 months |  | 64.0 ± 22.8 ^a^  20.1 ± 18.8  24.7 ± 20.4  20.2 ± 15.6 |  | 65.5 ± 25.6 ^a^  19.6 ± 17.7  22.7 ± 21.6  24.4 ± 20.1 |  |

* represents intergroup differences *(P* <0.05, ANCOVA adjusted for antimicrobial usage and corresponding variable at baseline). ^a^ represents intragroup differences in comparison with the other follow-up points (*p* <0.001 ANOVA for repeated measures including Bonferroni correction). Abbreviations: PPD, Probing Pocket depth; CAL, Clinical Attachment Level; REC, recessions; BOP, Bleeding On Probing; PISA, Periodontal Inflamed Surface Area and see Table 1.

*Table S2.* Effect of periodontal therapy on the markers of metabolic syndrome (MetS). Values are means ± standard deviation.

|  | |  | **BPT** |  | **BPT+AM** |
| --- | --- | --- | --- | --- | --- |
|  |  |  | N=56 |  | N=54 |
| WC (cm) | Baseline  3 months  6 months  12 months |  | 92.0 ± 12.9  90.2 ± 12.7  91.1 ± 12.3  93.1 ± 11.7 ^ab^ |  | 91.6 ± 11.7  91.7 ± 11.8  91.6 ± 11.6  92.7 ± 11.7 ^ab^ |
| Blood pressure |  |  |  |  |  |
| SBP (mmHg) | Baseline  3 months  6 months  12 months |  | 134.8 ± 20.0  132.5 ± 17.5 ^c^  132.1 ± 17.6 ^c^  132.1 ± 19.8 ^d^ |  | 138.9 ± 20.0  133.5 ± 17.3 ^c^  130.8 ± 17.1 ^c^  133.5 ± 19.7 ^d^ |
| DBP (mmHg) | Baseline  3 months  6 months  12 months |  | 79.5 ± 12.1  80.6 ± 11.7  80.0 ± 11.8  79.7 ± 12.5 |  | 81.0 ± 11.4  81.1 ± 10.7  80.7 ± 11.7  81.6 ± 10.6 |
| HDL (mmol/L) | Baseline  3 months  6 months  12 months |  | 1.39 ± 0.46  1.40 ± 0.41  1.38 ± 0.43  1.38 ± 0.41 |  | 1.37 ± 0.39  1.38 ± 0.48  1.37 ± 0.43  1.40 ± 0.42 |
| Tryglicerides (mmol/L) | Baseline  3 months  6 months  12 months |  | 1.71 ± 1.09  1.17 ± 0.74 ^c^  1.27 ± 0.96 ^c^  1.35 ± 0.96 ^d^ |  | 1.59 ± 1.25  1.23 ± 1.02 ^c^  1.13 ± 0.75 ^c^  1.28 ± 1.06 ^d^ |
| Glucose (mmol/L) | Baseline  3 months  6 months  12 months |  | 5.69 ± 0.64  5.66 ± 0.56  5.66 ± 0.63  5.64 ± 0.68 |  | 5.60 ± 0.56  5.43 ± 0.46  5.47 ± 0.49  5.46 ± 0.75 |

* Based on intention-to-treat analysis. ^a^*p*_adj_ <0.05 in comparison to 3 months (ANOVA for repeated measures + Bonferroni). ^b^*p*_adj_ <0.05 in comparison to 6 months (ANOVA for repeated measures + Bonferroni). ^c^*p*_adj_ <0.01 in comparison to baseline (ANOVA for repeated measures + Bonferroni). ^d^*p_a_*_dj_ <0.05 in comparison to baseline (ANOVA for repeated measures + Bonferroni). Smoking was a significant covariate associated with triglycerides *p*_adj_ <0.003. Abbreviations: WC, waist circumference; SBP, systolic blood pressure; DBP, diastolic blood pressure; HDL, high-density lipoproteins and see Table 1.

*Supplemental Table S3*. Distribution of individuals with and without diagnosis of metabolic syndrome (MetS). Values are number of subjects (%).

|  | **BPT** | | |  | **BPT+AM** | |  | **Total** | |
| --- | --- | --- | --- | --- | --- | --- | --- | --- | --- |
|  |  | MetS+ | MetS- |  | MetS+ | MetS- |  | MetS+ | MetS- |
| Baseline | | 14 (25.0) | 42 (75.0) |  | 16 (29.6) | 38 (70.4) |  | 30 (27.2) | 80 (72.8) |
| 3 months | | 10 (18.2) | 45 (81.8) |  | 6 (12.0) | 44 (88.0) ^c^ |  | 16 (15.2) | 89 (84.8) ^a^ |
| 6 months | | 11 (21.2) | 41 (78.8) |  | 5 (10.6)^c^ | 42 (89.4) ^d^ |  | 16 (16.2) | 83 (83.8) ^b^ |
| 12 months | | 13 (25.5) | 38 (74.5) |  | 8 (16.7) | 40 (83.3) ^e^ |  | 21 (21.2) | 78 (78.8) |

Based on per-protocol analysis. ^a^*p* = 0.011, ^b^*p* = 0.012, ^c^*p* = 0.022, ^d^*p* = 0.012, ^e^*p* = 0.039 (Mc Nemar test, *p* values ≤ 0.017 were considered statistical significant, to correct for 3 pairwise comparisons). Diagnosis of metabolic syndrome: presence of >3 of the following parameters: waist circumference >102 cm in men or >88 cm in women, triglycerides >1.7 mmol/L, HDL-cholesterol <1.03 mmol/L in men or <1.29 mmol/L in women, blood pressure >130/85 mmHg, fasting glucose >5.6 mmol/L. Abbreviations: MetS+, patients with diagnosis of metabolic syndrome; MetS-, patients without diagnosis of metabolic syndrome and see Table 1.

*Supplemental Table S4.* Numbers of individuals with and without diagnosis of metabolic syndrome (MetS) distributed per treatment protocol.

Values are number of subjects .

|  | No of subjects* | No of subjects* | |  | | | | No of subjects* | | |
| --- | --- | --- | --- | --- | --- | --- | --- | --- | --- | --- |
|  | **Total population** | **MetS+ (BPT+AM; BPT)** | |  | | | | **MetS- (BPT+AM; BPT)** | | |
| Baseline | 110 | 30 (16; 14) | | |  | | | 80 (38; 42) | | |
|  |  | MetS+  (BPT+AM; BPT) | MetS-  (BPT+AM; BPT) | | |  | MetS+  (BPT+AM; BPT) | | MetS-  (BPT+AM; BPT) |  |
| 3 months | 105 | 11  (4; 7) | 18  (11; 7) | | |  | 5  (2; 3) | | 71  (33; 38) |  |
| 6 months | 99 | 12  (4; 8) | 16  (10; 6) | | |  | 4  (1; 3) | | 67  (32; 35) |  |
| 12 months | 99 | 16  (6; 10) | 13  (10; 3) | | |  | 5  (2; 3) | | 65  (30; 35) |  |

* Numbers are based on per-protocol analysis. Diagnosis of metabolic syndrome: presence of >3 of the following parameters: waist cirucumference >102 cm in men or >88 cm in women, triglycerides >1.7 mmol/L, HDL-cholesterol <1.03 mmol/L in men or <1.29 mmol/L in women, blood pressure >130/85 mmHg, fasting glucose >5.6 mmol/L. Abbreviations: MetS+, patients with diagnosis of metabolic syndrome; MetS-, patients without diagnosis of metabolic syndrome.

**Figure S1**

Changes for the MetS parameters during 12 months follow-up for MetS+ (N=30) and MetS- (N=80) periodontitis patients at baseline. Changes for waist circumference (**A**), triglycerides (**B**), HDL-cholesterol (**C**), systolic blood pressure (**D**), diastolic blood pressure (**E**) and glucose (**F**) are presented. Error bars represent standard errors of means. *P_adj_* for intragroup differences are obtained from analysis of ANOVA for repeated measures adjusted for gender, age and smoking. MetS = Metabolic Syndrome.
